# A RARE PRESENTATION OF PEMPHIGUS VULGARIS AS MULTIPLE PUSTULES

**DOI:** 10.4103/0019-5154.70675

**Published:** 2010

**Authors:** Yang Yang, Min Lin, Su Jiang Huang, Chen Min, Wan Qing Liao

**Affiliations:** *From the Department of Dermatology, Guangdong Provincial Crops Hospital, Chinese people’s Armed Police Forces, Guangzhou, China*; 1 *Department of Dermatology, ChangZheng Hospital, Second Military Medical University, Shanghai, China. E-mail: liaowanqing@sohu.com*

**Figure 1:**
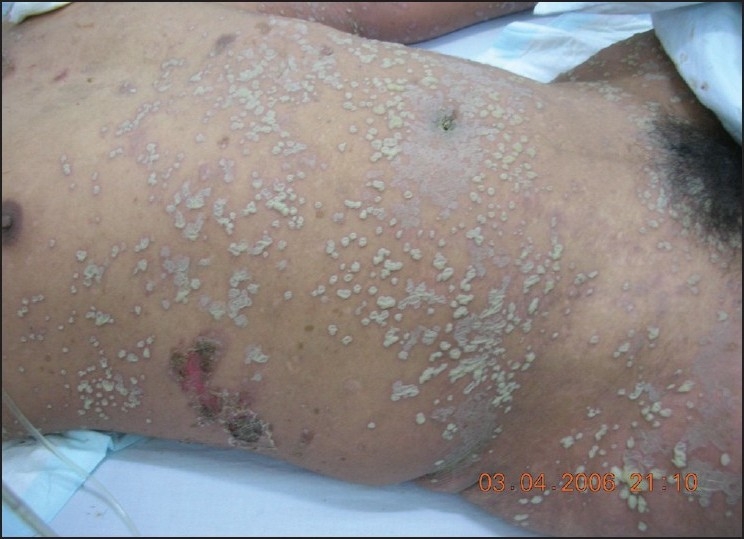
Soybean-like multiple pustules all over body

**Figure 2:**
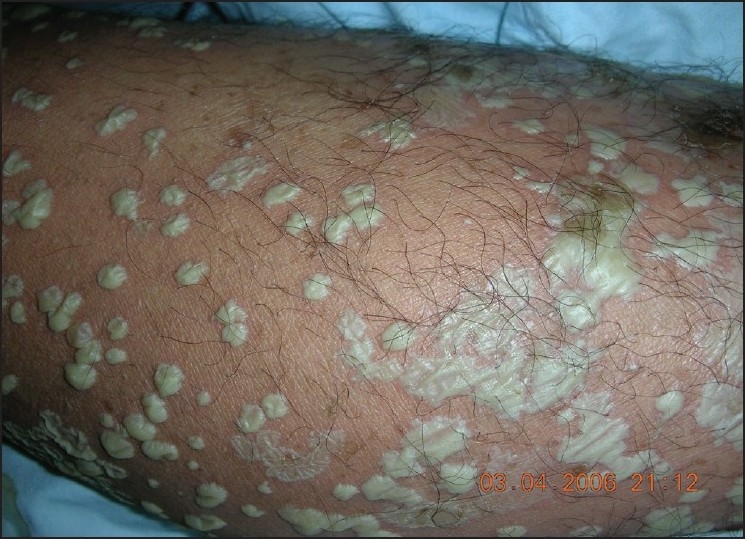
Multiple pustules on the right elbow, some pustules connects to pustules lakes

A 34-year-old, previously healthy man presented with pustule on the site of right costal part for one week. Pustules gradually spreaded all over the trunk, buttocks, and limbs with pruritus [Figures 1 and 2]. The patient developed fever up to 39°C as well as chills the day before being admitted to our hospital. He had received antibiotics therapies at other clinics and did not receive any response. On admission, his body temperature was 39°C, blood pressure was 90/60 mmHg. Examination revealed multiple dense pustules all over the trunk, buttocks, and limbs. Pustules are tense and some of which measure up to 3 × 2cm. Owing to position, liquor puris was located on the inferior surface of pustule, appeared like demilune, Some pustules coalesced to form lakes of pus, some dried, and some breaked and left red moist erosions. Nikolsky’s sign was not present. Mucous membrane lesions of eyes, oral cavity, and genitals were not found. The rest results of physical examination did not show anything abnormal.

**Figure 3:**
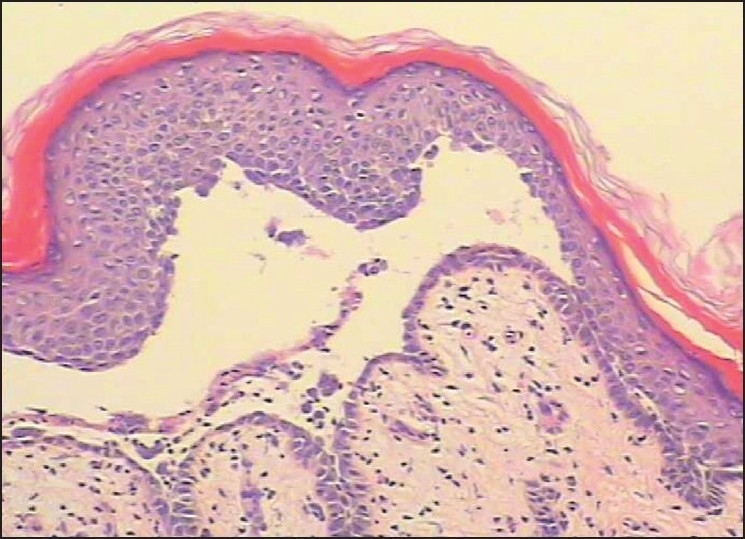
Pustules in epidermis (H and E stain, ×100)

**Figure 4:**
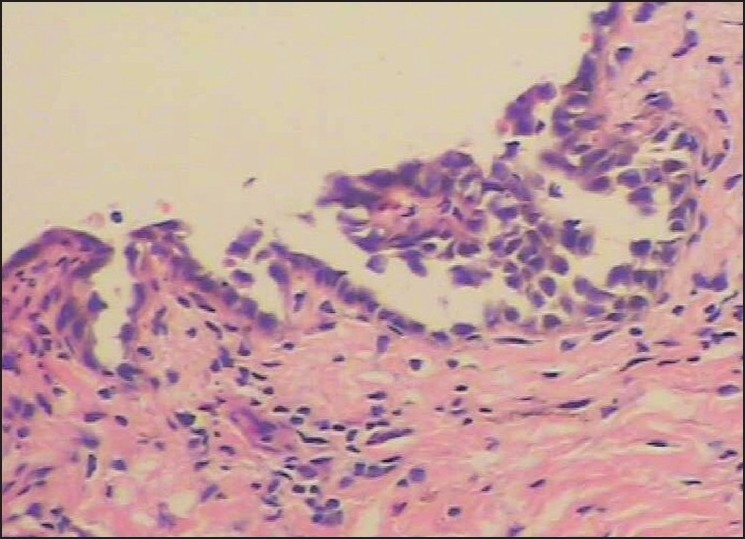
Acantholytic cell at the bottom of pustules (H and E stain, ×100)

**Figure 5:**
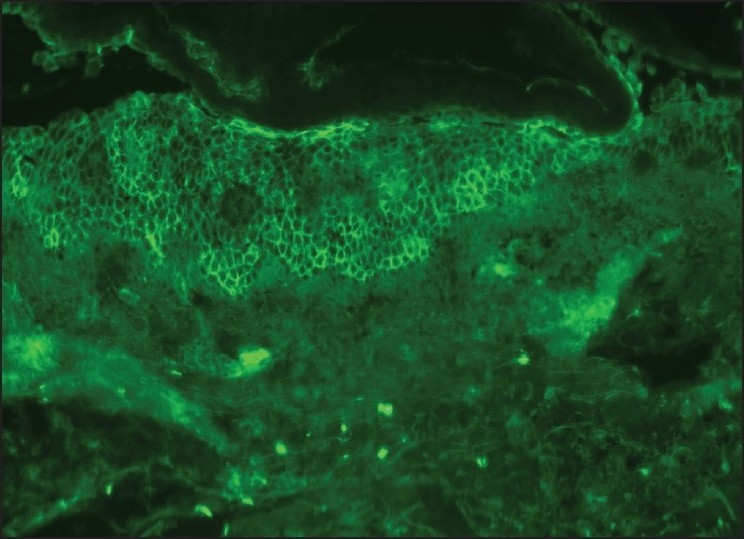
Intercellular reticulate deposition of IgG and weak intercellular deposit in the basal epithelium by direct immunofluorescence analysis

**Figure 6:**
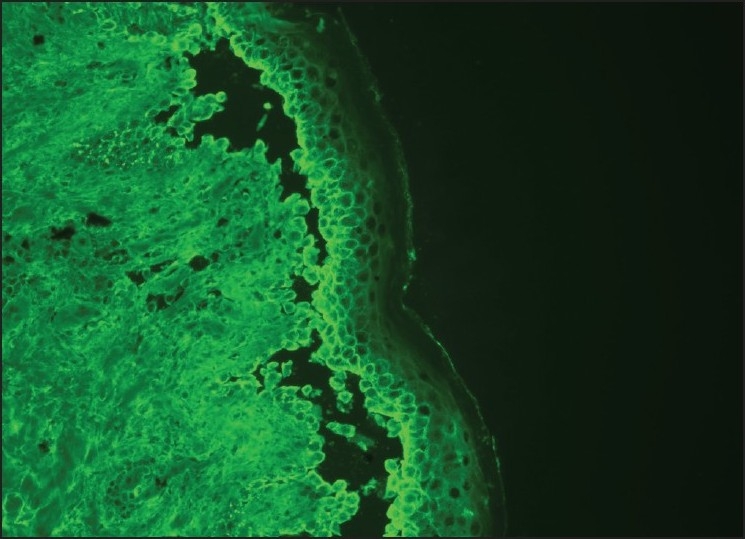
Intercellular deposits of C3 in the basal epithelium by direct immunofluorescence analysis

Laboratory studies revealed a white blood cell count of 10.8×10^9^/L with granulocytosis (94.1%), and C reactive protein was raised (+); Bacteriologic culture of the pus was negative; antibodies to HCV was positive; antistreptolysin O, rheumatoid factor, ENA polypeptide antibody and HIV- antibody were negative. USG examination showed liver, guts, spleen, and pancreas were normal; the evaluation of chest X ray was normal. Skin biopsy showed spinous layer solution fissure and suprabasal intraepidermal blisters [Figure 3]. Blisters cells contained some acantholytic cells: corium papilla upward accrementition in the bottom of blisters, covered with monolayer basal cell to form villus, part of bottom of blister epidermal cell regeneration ambo aliquation, dermis [Figure 4]. The superficial dermis has a mild, mixed inflammatory infiltration. Direct immunofluorescence demonstrated intercellular IgG and C_3_ deposits throughout the epidermis in and around lesions [Figures 5 and 6] IgA, IgM, C4 and C1q were absent.

After admitted, the patient was first given ceftriaxone sodium 3.0g/day, combined with clindamycin 600mg/day and levofloxacin 200 mg/day for 2 days. The amount of pustules still increased day by day. Then with the patient’s consent for systemic corticosteroid therapy, we started treatment with methyprednisolone (160 mg/day or 2.5mg/kg) plus γ-globulin (7.5g/day). Within 2 days, pustules formation decreased and lesions started regression. Then, we reduced the dosage of methyprednisolone by 40mg/day till 60mg/day and maintained for 3 days. Finally, the dose was diminished to 40 mg/day till the patient was discharged. Meanwhile, supportive treatment including diammonium glycyrrhizinate, IVIg, nursing care, and changing dressings to the skin was employed. After 45 days, most of the lesions dried and started healing and the patient was discharged and advised to take prednisone 30 mg/day. After 2 months, the patient reduced the dose to 20 mg/day without instruction and soon symptoms rebounded, Now, symptoms were controlled by boosting dosage to 60mg/day, and the patient is still in the follow-up period.

Pemphigus vulgaris is a clinically common disease,[1–3] however, pemphigus vulgaris with pustules bullosa is infrequent. Japanese expert Matsuo and his colleagues reported four cases of pemphigus foliaceus patients with pustule.[4] The highlight of diagnosis of pemphigus vulgaris, including lusitropic bullae, Nikolsky’s sign is present, blisters break easily and producing painful erosions, along with mucous membrane lesions, characteristic histopatologic changes, acantholysis in the epidermis, direct immunofluorescence examination shows IgG and C3 deposition among epidermal cells.

This patient had special lesions, Pustules bullosa was the initial manifestation, blisters were firm, tense and did not broke easily, Nikolsky’s sign was absent, pustules existed longer, and most pustules dried spontaneously with blister walls intact, few red moist erosions was found. In general, such clinical conditions would always be taken for blistering dermatoses such as crusted tetter, IgA pemphigus or Sneddon–Wilkinson disease. However, finally, we made a definite diagnosis of pemphigus vulgaris by histopathology and immunofluorescence examination, and the patient responded well to steroid therapy, we did not find the same or similar case report published elsewhere.

During differential diagnoses, six types of diseases, including crusted tetter with bullae, IgA pemphigus, impetigo herpetiformis, rash with pustulosis with acute exanthesis, Sneddon-Wilkinson disease, and generalized pustular psoriasis, should be kept in mind. Crusted tetter with bullae is a contagious infection of the skin with purulence in children, mostly caused by coagulase-positive *Staphylococcus aureus*. Bullous lesions usually occur on the face, trunk, extremities, buttocks, or perineal regions. Bullae are thin-roofed flaccid or soon become flaccid of various sizes, containing clear yellow fluid at the beginning and become turbid and pyoid after one day. Pus deposit on the bottom of bullae to form characteristic lunate phenomenon of empyema. Histological examination reveals blisters in the subcorneal or granular region; polymorphonuclear cells and fibrins are usually present within the vesicle; Cocci may be observed on gram stain. Bacterial culture of pus yields *staphylococci*. Antibiotics therapy is effective in controlling this.

IgA pemphigus is a clinically rare disease, which always affects the site of wrinkle of the body. Old women are at higher risk compared with other groups of people. Patients develop vesicles or pustules on normal or erythematous skin. Lesions are widespread and apt to coalesce. Direct immunofluorescence detects predomainantly IgA deposits at the cell surfaces of the epidermis.

Acute generalized exanthematous pustulosis is a disease manifested by the diffuse eruption of follicular sterile pustules. Most of the cases are induced by drugs. Skin biopsy reveals subcorneal pustules containing leukocytes and a mixed perivascular inflammatory infiltrate in the dermis.

Sneddon–Wilkinson disease mostly occurs in middle-aged women; generally, it is innocuous; armpit and groin are usually involved; however, face is not generally involved. The classic lesion has been described as a “half-half” blister, in which purulent fluid accumulates in the lower half of the blister. And the upper part fluid shows clear. Direct and indirect immunofluorescence studies are typically negative. Generalized pustular psoriasis is an uncommon form of psoriasis consisting of widespread pustules in an erythematous background. These patients are characterized by having a history of psoriasis and may have residual lesions of psoriasis vulgaris. Histological epidermal changes are similar to those of psoriasis vulgaris, with parakeratosis and elongation of rete ridges. There is a dermal and epidermal infiltration, and spongiform pustule of Kogoj is present.

Systemic corticosteroids therapy is mainly used for the principal management of pemphigus vulgaris. Initially, we use high dose of steroid to control symptom, and then gradually reduced steroid dosage. In this way, we rapidly improved the patient with good control of the disease. The diagnosis and treatment of this case demonstrate that histopathology and immunopathogenesis play important roles in the diagnosis of bullae and pustule diseases. The case we reported herein is a rare type of pemphigus vulgaris. Although we failed to demonstrate that it is a new subtype of pemphigus vulgaris, it is obvious that pathogenesis and pathophysiology still need further investigation.
